# Development of a hexavalent recombinant protein vaccine adjuvanted with Montanide ISA 50 V and determination of its protective efficacy against acute toxoplasmosis

**DOI:** 10.1186/s12879-020-05220-2

**Published:** 2020-07-10

**Authors:** Esra Atalay Şahar, Hüseyin Can, Sultan Gülçe İz, Aysu Değirmenci Döşkaya, Mina Kalantari-Dehaghi, Remziye Deveci, Adnan Yüksel Gürüz, Mert Döşkaya

**Affiliations:** 1 Present address: Department of Parasitology, Vaccine Research and Development Laboratory, Faculty of Medicine, Ege University, Bornova, 35100 İzmir, Turkey; 2 Department of Molecular Biology, Faculty of Science, Ege University, İzmir, 35100 Bornova, Turkey; 3 Department of Biotechnology, Ege University Faculty of Science, Bornova, 35100 İzmir, Turkey; 4 Department of Bioengineering, Ege University Faculty of Engineering, Bornova, 35100 İzmir, Turkey; 5 Department of Dermatology, University of California Irvine, Irvine, CA 92617 USA

**Keywords:** *Toxoplasma gondii*, Recombinant protein vaccine, Hexavalant, Adjuvant, Montanide ISA 50 V

## Abstract

**Background:**

*Toxoplasma gondii* is an obligate intracellular parasite that can infect almost all warm-blooded animals, avian species and humans. Toxoplasmosis is asymptomatic in healthy individuals, whereas it may lead to death in immune suppressed or deficient patients. A vaccine against *T. gondii* is required to prevent consequences of the infection. The aim of this study is to generate a multivalent recombinant protein vaccine against *T. gondii.*

**Methods:**

49 previously discovered antigenic proteins of *T gondii* were evaluated by their expression level in *E. coli* and by comprehensive bioinformatics analyses to determine antigenic epitopes. Based on these analyses, six vaccine candidate proteins were selected to generate a hexavalent recombinant protein vaccine adjuvanted with Montanide ISA 50 V. Humoral and cellular immune responses were determined by flow cytometry and ELISA. Vaccinated mice were challenged with *T. gondii* Ankara strain tachyzoites.

**Results:**

In mice vaccinated with hexavalent vaccine, strong total IgG (*P* < 0.0001) and IgG2a (*P* < 0.001) responses were induced compared to controls, the ratio of CD4^+^ and CD8^+^ T lymphocytes secreting IFN-γ increased, and significantly higher extracellular IFN-γ secretion was achieved compared to the controls (*P* < 0.001). The survival time of the vaccinated mice increased to 8.38 ± 2.13 days which was significantly higher than controls (*P* < 0.01).

**Conclusions:**

Altogether, these results show that the hexavalent vaccine which is developed for the first time against *T. gondii* induced strong and balanced Th1 and Th2 immune responses as well as conferred significant protection against challenge with lethal toxoplasmosis in murine model.

## Background

*Toxoplasma gondii* is an obligate intracellular parasite that causes toxoplasmosis in all warm blooded animals and humans. It is reported that one third of the world’s population is estimated to be infected with *T. gondii*. *T. gondii* usually causes an asymptomatic infection in healthy people, but can be life-threating in immune-compromised patients (organ transplant recipients, Acquired Immunodeficiency Syndrome patients, and cancer patients). Congenital toxoplasmosis may cause abortion, neonatal death or foetal abnormalities in the foetus [1]. Farm animals such as sheep, goats, and pigs have also been shown to be susceptible to *T. gondii* infection. Toxoplasmosis infection in farm animals causes significant economic losses as a result of prenatal death, abortion, and neonatal death. Moreover, *T. gondii* is linked to mental disorders and may affect human behaviour, personality, and other phenotypic traits [2].

Cats and other felines are the definitive hosts of *T. gondii* [3]. In the life cycle of *T. gondii* the main infective forms are tissue cysts (containing bradyzoites) and oocysts (containing sporozoites). The infection sources for humans are consumption of vegetables, fruits or water contaminated with faeces of infected cats containing oocysts and raw or undercooked meat contaminated with tissue cysts [4]. For these reasons, development of a safe and protective vaccine against *T. gondii* infection that can be used in animals and humans has utmost importance.

Recombinant protein vaccines are safe and efficient, and have a great potential for prevention or eradication of diseases. One of the most important issues during a recombinant protein vaccine development is the selection of the vaccine candidate antigen(s) [5]. Antigens to be used in vaccine formulations against toxoplasmosis should actively induced strong immune response, produce long-lasting immunity, and be antigenic in each stage of the parasite [6]. In our study group’s previous studies, we used protein microarray containing 2870 candidate exon products of *T. gondii* to screen well-characterized sera from acute and chronic toxoplasmosis human cases and murine model infected orally with oocysts or tissue cysts. During these studies, screening human sera prioritized 240 antigenic proteins and screening these 240 antigens with murine sera prioritized 49 proteins based on their immunogenicity [6–8].

In this study, we selected six proteins based on their antigenic epitopes using bioinformatics and protein expression level in *E. coli*. Thereafter, we developed a hexavalent recombinant protein vaccine adjuvanted with Montanide ISA 50 V and administered to a murine model to determine its immunogenicity and protection efficiency against lethal toxoplasmosis.

## Methods

### Animals

6–8 week old female healthy Swiss outbred mice were obtained from the Bornova Veterinary Control Institute Animal Production Facility and used during the experiments. Animals were housed under standard and suitable conditions. Specifically rooms had ambient temperature and humidity, adequate light cycle, and diet was specific for each animal type. All animals were checked for humane endpoints every day such as rapid weight loss more than ~ 20% of gross body weight, inability to assess water or food, or loss of skin elasticity indicative of dehydration. In any of these circumstances, we pre-euthanized the animals with ketamine hydrochloride (2 mg/kg) and 2% xylazine (3 mg/kg) and then euthanized with cervical dislocation.

### Determination of vaccine candidate antigens

Based on the data obtained from previous studies [6–8], 49 proteins were selected based on their immunogenicity in humans and murine sera (Table 1). In order to determine the vaccine candidate antigens, expression levels of these proteins were analysed using western blot and moreover, a comprehensive bioinformatics analyses was performed.

**Table 1:** The plasmids encoding selected 49 *T. gondii* antigenic proteins

ToxoDB^a^ name	Plasmid name	ToxoDB^a^ name	Plasmid name
TGME49_047370_4	pA1	TGME49_034360_1	pB4
TGME49_026020_8	pB1	TGME49_113440_1	pC4
TGME49_053730_9	pC1	TGME49_013390_6	pD4
TGME49_061020_40	pD1	**TGME49_073130_2**	**pE4**
TGME49_047390_4	pE1	TGME49_075490_12	pF4
TGME49_024710_6	pF1	TGME49_114850_1	pG4
TGME49_104950_2	pG1	TGME49_047540_5	pH 4
TGME49_086450_1	pH 1	TGME49_110780_1	pA5
TGME49_021710_4	pA2	TGME49_026110_8	pB5
TGME49_121520_3	pB2	TGME49_093000_2	pC5
TGME49_037880_1	pC2	TGME49_078100_1	pD5
TGME49_093730_6	pD2	TGME49_092280_1	pE5
TGME49_015980_1	pE2	TGME49_026380_1	pF5
TGME49_001840_1	pF2	TGME49_090680_8	pG5
TGME49_039440_1	pG2	TGME49_005360_17	pH 5
**TGME49_019310_8**	**pH 2**	TGME49_109590_1	pA6
TGME49_027280_1	pA3	TGME49_114500_1	pB6
TGME49_003310_1	pB3	TGME49_055180_14	pC6
TGME49_048540_4	pC3	**TGME49_055180_7**	**pD6**
TGME49_100120_9	pD3	**TGME49_025340_13**	**pE6**
TGME49_095650_3	pE3	TGME49_022370_1	pF6
TGME49_051630_2	pF3	TGME49_026510_1	pG6
TGME49_054720_1	pG3	**TGME49_013390_5**	**pH 6**
TGME49_005360_12	pH 3	TGME49_095700_1	pA7
**TGME49_058660_1**	**pA4**		

^a^ToxoDB: http://www.toxodb.org/toxo/

Bold written plasmids are the antigens selected for hexavalent vaccine development

### Expression levels of 49 proteins in *E. coli*

The gene sequences of the 49 different genes were accessible from the *Toxoplasma* Genomic Resource (http://www.toxodb.org/toxo/). The plasmids expressing these 49 recombinant proteins were constructed as previously described [6–8]. Thereafter, the plasmids were cloned into chemically competent *Escherichia coli* (*E. coli*) BL21 Star (DE3) pLysS cells according to the manufacturer’s protocol (Invitrogen, USA).

*E. coli* BL21 Star (DE3) pLysS cells containing 49 different plasmids were incubated at 37 °C with shaking at 225 rpm for 16–24 h until the optical density (OD600) reached an absorbance of 0,4 ng/μL. Then, recombinant protein expression was induced with 0.5 mM IPTG (*isopropyl-D- thiogalactopyranoside*) and the bacterial cultures were incubated for 4 h, 37 °C with shaking at 225 rpm. Next, cell cultures were harvested by centrifugation at 5000×g for 10 min. The pellets were homogenized with lysis buffer [0.1% Triton X-100, 50 mM Tris-Cl and 0.3 M NaCl (pH: 7.4)] and were freeze-thawed 3 times. The homogenates were incubated on a rotator at room temperature for 15 min and then were centrifuged at 30.000×g for 20 min.

The supernatants were incubated with 1 ml Ni-NTA Superflow beads (Qiagen, USA) for 30 min with shaking at 100 rpm. At the end of incubation, the suspension was centrifuged at 4000 rpm for 1 min and the supernatant was discarded. Then, the Ni-NTA beads were washed with 50 mM Tris-Cl and 0.3 M NaCl and 25 mM imidazole (pH: 7.4) for 30 min with shaking at 100 rpm. Thereafter, the suspension was centrifuged at 4000 rpm for 1 min and the supernatant was discarded. Next, Ni-NTA beads were incubated with 50 mM Tris-Cl and 0.3 M NaCl and 0.5 M imidazole (pH: 7.4) for 30 min with shaking at 100 rpm. Finally, the suspension was centrifuged at 4000 rpm for 1 min and supernatants were analysed with Western blot as described below to determine the expression level of recombinant proteins. The most abundantly expressed recombinant proteins were selected for vaccine development.

### Bioinformatics analyses of 49 recombinant proteins for antigen mapping and glycosylation

The protective immune response against toxoplasmosis is conferred by mainly by cellular immune response and through humoral immune response [6, 9]. In addition, the expression of 49 recombinant proteins of *T. gondii* will be performed in *E. coli*. *T. gondii* is a eukaryotic obligate intracellular parasite. Protein post-translational modifications are common events in most eukaryotes, such as glycosylation. Glycosylation of peptide effects protein immunogenicity and major histocompatibility complex (MHC) binding [10]. Thus, apart from the determination of the expression levels, 49 proteins will analysed by bioinformatics tools to determine the presence of antigenic epitopes as well as glycosylation sites. At the end of the bioinformatics analyses, we aimed to select the proteins that have cellular and humoral immune response inducing epitopes with the least glycosylation sites.

Since *T. gondii* is an intracellular parasite, immune-mediated protection against toxoplasmosis is mainly conferred by interaction between CD8^+^ T cells and MHC Class I and CD4^+^ T cells and MHC Class II [11–14]. Antigenic sites recognized by CD8^+^ T cells and CD4^**+**^are peptides containing 8 to 10 and 15 amino acids associated with the MHC Class I (MHC-I) and MHC-II molecules, respectively [12–14]. Predicted epitopes of CD8^+^ T cells and CD4^**+**^ T cells that bind to MHC-I and MHC-II inside 49 proteins were investigated using the Immune Epitope Database and Analysis Resource (IEDB) (http://tools.immuneepitope.org/mhci; http://tools.iedb.org/mhcii/) [13–18]. The potential linear B-cell epitopes of 49 antigenic proteins were also analysed by Support Vector Machine (SVM) which has been utilized by combining the Tri-peptide similarity and Propensity scores (SVMTriP; http://sysbio.unl.edu/SVMTriP/prediction.php) [19].

In many eukaryotic pathogens, proteins may have N- and O-linked glycosylation sites through post translational modification which may affect their interaction with their host organisms. Protein glycosylation is common in *T. gondii* as it is in other eukaryotes [20]. In this study, *E. coli* was used as the expression system and we aimed to select the vaccine candidate proteins among the 49 recombinant proteins with least glycosylation sites in predicted antigenic epitopes. The potential N-glycosylation and O-glycosylation sites of 49 antigenic proteins were analysed using NetNGlyc 1.0 Server (http://www.cbs.dtu.dk/services/NetNGlyc/) NetOGlyc 4.0 Server (http://www.cbs.dtu.dk/services/NetOGlyc/) [21].

### Expression and purification of vaccine candidate 6 antigenic proteins

According to protein expression levels and bioinformatics results, 6 proteins were [pH 2 (TGME49_019310_8), pA4 (TGME49_058660_1), pE4 (TGME49_073130_2), pD6 (TGME49_055180_7), pE6 (TGME49_025340_13), pH 6 (TGME49_013390_5)] were selected as vaccine candidate. Thereafter, *E. coli* BL21 Star (DE3) pLysS cells containing the pH 2, pA4, pE4, pD6, pE6, and pH 6 stocks were inoculated into individual 500 ml LB broth medium supplemented with ampicillin (100 μg/ml) and incubated overnight at 37 °C with 225 rpm shaking. Next day, the overnight cultures were inoculated into the bioreactor (Bioflo 110, New Brunswick, USA) containing 7.5 L enrichment medium supplemented with ampicillin (100 μg/ml). The dissolved oxygen and pH levels were maintained at 40–60 and 7.0 ± 0.4 with vigorous mixing (400 rpm) at 37 °C until OD600 reached 0.4. Then, the cell cultures were induced at a final concentration of 0.5 mM IPTG and incubated for 4 h at 37 °C.

The cells were centrifuged at 5000×g for 10 min and the pellet was resuspended with 300 ml pre-chilled loading buffer (50 mM Tris-Cl, 0.3 M NaCl, pH 7.5) and homogenized with a blender for 10 s (Waring, USA). Then, the homogenized cells were disrupted twice using a microfluidizer processor (Microfluidics M-110 L Pneumatic, USA) at a low temperature under internal pressure of 18,000 psi and centrifuged at 30,000 g for ½ h at 4 °C. The homogenates were centrifuged at 30,000×g for 30 min at 4 °C. The clarified supernatants were filtered using 0.45 μm pore filter (Corning, USA). The filtered samples were purified with AKTA Fast Protein Liquid Chromatography (FPLC) system, controlled by UNICORN™ software (GE Health, USA), using 5 ml HiTrap Ni^2+^ Chelating HP column (GE Health, USA). Approximately 100 ml filtered supernatant was loaded to the HiTrap Ni^2+^ Chelating HP column. After binding, the column was washed with buffers containing increasing concentrations of imidazole (50 mM, 100 mM, 250 mM). The recombinant proteins (rH2, rA4, rE4, rD6, rE6, and rH6) were eluted by raising the imidazole concentration to 0.5 M. Purity and identity of the purified proteins were analysed by 12% (sodium dodecyl sulfate-polyacrylamide gel electrophoresis) SDS-PAGE and Western Blot analysis and concentrated with Vivaspin 20 (Sartorius, Germany).

The proteins were further purified by FPLC on a Superdex 200/10–300 GL [100000–600,000 Molecular weight cut-off (MWCO)] column (GE Health, USA) to remove excess endotoxin. The subsequent protein fractions were pooled, concentrated and quantitated by Bradford method (Pierce, USA).

### SDS-PAGE and Western blot analysis

To observe the expression levels, purity and immunoreactivity, proteins were separated by 12% sodium dodecyl sulfate-polyacrylamide gel (SDS-PAGE). The separated proteins were transferred to polyvinylidene difluoride (PVDF) transfer membrane (Immobilon-P, Millipore, MA), blocked by 6.25% non-fat dry milk for 1 h at room temperature. The membranes were probed with a 1/3333 dilution of monoclonal anti-polyhistidine antibody (Sigma-Aldrich, USA) for 1.5 h at room temperature. Then the membranes were probed with a 1/3333 dilution of alkaline phosphatase-conjugated goat anti-mouse IgG (H + L) antibody (Sigma-Aldrich, USA) for 1 h at room temperature. The blot was developed with diethanolamine buffer (10% Diethanolamine, 4 M HCl pH: 9.8, 0.5 mM MgCl2•6H2O) containing 4.3% 5-bromo-4-chloro-3-indolyl phosphate (BCIP) diluted in dimethylacetamide, 4.1% Nitro-BT diluted in 70% (v/v) dimethylformamide (Applichem, Germany).

### Determination and removal of endotoxin from purified recombinant proteins

The amount of endotoxin in the purified vaccine candidate 6 recombinant proteins were determined with Limulus Ameobocyte Lysate (LAL) Gel-Clot test using Pyrotell single test vials according to the manufacturer’s protocol (Cape Cod Inc., USA). The test sensitivity was 0.25 Endotoxin Units (EU)/mL. *E. coli* O113:H10 control standard endotoxin (Cape Cod Inc., USA) was used for positive control and LAL reagent water was used for negative control. The concentration of endotoxin-depleted, purified recombinant protein samples were calculated with Bradford method (Pierce, USA) and stored at − 80 °C until use.

### Vaccination and *T. gondii* challenge infection

6–8 weeks old female Swiss Webster outbred mice were randomly divided into four groups, each group consisting of eleven mice (n:11). Mice were vaccinated intraperitoneally (i.p.) twice at 3 weeks intervals. Vaccines and control groups as shown in Table 2. The first group was immunized with hexavalent (6 antigens) recombinant proteins adjuvanted with Montanide ISA 50 V (Seppic, France) prepared according to the manufacturer’s protocols. Montanide ISA 50 V was used as adjuvant due to its efficiency in inducing both humoral and cellular immune response. Three groups were considered as control; one control group was administered only with hexavalent recombinant proteins without Montanide ISA 50 V adjuvant. The adjuvant control group was inoculated with only Montanide ISA 50 V [100 μl Montanide ISA 50 V adjuvant + 100 μl Phosphate buffered saline (PBS)] and the last control group was inoculated only PBS (200 μl PBS). Tail bleeds were performed 3 weeks after each vaccination.

**Table 2:** Vaccine and control groups used during vaccination of mice

Vaccine and control groups (n: 11)	Dose	
	Prime dose (day 0)	Boost dose (day 21)
**PBS**	100 μl PBS / i.p.	100 μl PBS / i.p.
**Montanide ISA 50 V**	100 μl Montanide (+) 100 μl PBS	100 μl Montanide (+) 100 μl PBS
**Hexavalent recombinant protein mixture**	Contains 6 different recombinant proteins [rH2 (+) rA4 (+) rE4 (+) rD6 (+) rE6 (+) rH6] in PBS. The amount of each protein is 20 μg in 200 μl PBS.	Contains 6 different recombinant proteins [rH2 (+) rA4 (+) rE4 (+) rD6 (+) rE6 (+) rH6] in PBS. The amount of each protein is 20 μg in 200 μl PBS.
**Hexavalent recombinant protein mixture (+) Montanide ISA 50 V**	Contains 6 different recombinant proteins [rH2 (+) rA4 (+) rE4 (+) rD6 (+) rE6 (+) rH6] in PBS. The amount of each protein is 20 μg in 100 μl PBS (+) 100 μl Montanide ISA 50 V	Contains 6 different recombinant proteins [rH2 (+) rA4 (+) rE4 (+) rD6 (+) rE6 (+) rH6] in PBS. The amount of each protein is 20 μg in 100 μl PBS (+) 100 μl Montanide ISA 50 V

Nine weeks after first vaccination, eight mice from all four groups were challenged intraperitoneally with 1 × 10^5^ tachyzoites of *T. gondii* local strain called Ankara [22]. Thereafter, the infected mice were observed for the symptoms of toxoplasmosis such as loss of fur brightness and appetite and survival times were recorded on a daily basis.

### Detection of total IgG and IgG subclass antibody response using rec-ELISA

Determination of *T. gondii* specific total IgG, IgG1 and IGg2a antibodies in vaccinated mice were performed by recombinant Enzyme-Linked ImmunoSorbent Assay (Rec-ELISA) as described [23, 24]. In brief, each well of microplates (Nunc, USA) were coated with 100 μl of recombinant proteins solution (containing 5 μg/ml of each rH2, rA4, rE4, rD6, rE6 and rH6 in 1 × PBS) and incubated overnight at 4 °C. Next day, plates were washed 3 times with 300 μl PBS-T (0.05% Tween 20 in PBS) and then blocked with 5% nonfat dry milk containing 0.05% PBS-T for 2 h at room temperature. Mice sera were diluted to 1/100 with blocking buffer supplemented with *E. coli* lysate at a final concentration of 10 mg/ml protein to block *anti-E. coli* antibodies and incubated for 30 min at 37 °C. Then, the mouse sera were added to the wells in duplicate and incubated for 2 h at 37 °C with gentle shaking. After three washes with 300 μl PBS-T, the plates were incubated with 100 μl of anti-mouse IgG (Sigma-Aldrich, USA; diluted 1/2000 in 0.05% PBS-T), IgG1 (Jackson Immunoresearch, USA; diluted 1/1000 in 0.05% PBS-T) and IgG2a (Jackson Immunoresearch, USA; diluted 1/1500 in 0.05% PBS-T) conjugated with peroxidase for 1 h at room temperature. The plates were washed thrice with 300 μl PBS-T and incubated with 100 μl 3, 3′, 5, 5′ tetramethylbenzidine (TMB) substrate solution. The reaction was stopped by adding 75 μl of 2 N H_2_SO_4_ (Merck, USA) and the absorbance was measured at 450 nm using a microplate reader (Bio-Tek EL × 808, USA). Serum samples were accepted positive if the absorbance value (AV) of the serum sample exceeded the mean AV (+) 2× standard deviation (S.D.) of the negative control serum samples. Negative control serum samples are the day 0 serum samples of mice. Each plate contained *anti-*polyhistidine antibody (1:3333, Sigma-Aldrich, USA) probed control wells to determine the presence of His-tagged protein.

### Determination of extracellular cytokine by ELISA

To evaluated cytokine production, three mice from per group were euthanized 6 weeks after the prime vaccination and their spleens were removed. Single-cell suspensions of splenocytes were prepared as previously described [24, 25]. Aliquots of 5 × 10^5^ viable splenocytes in growth medium [1 × RPMI 1640 supplemented with 10% FCS (NBCS, HyClone, Thermo Fisher Scientific,USA), 2 mM l-glutamine (Gibco, Invitrogen,USA) penicillin (100 U/ml) and streptomycin (100 μg/ml) (Sigma-Aldrich, USA), 0.1 mM non-essential amino acid and 1 mM sodium pyruvate (Gibco, Invitrogen,USA)] were added to each well of 96 well round bottom plate (Greiner, Germany). Before stimulation of splenocytes, endotoxin-depleted purified rH2, rA4, rE4, rD6, rE6, and rH6 proteins were incubated with polymyxin B for 30 min at room temperature. As positive control, splenocytes were incubated with Concanavalin A (Sigma- Aldrich, Germany) at a final concentration of 10 μg/ml. As a negative control, only growth medium was used. Single cell suspensions were stimulated rH2, rA4, rE4, rD6, rE6, and rH6 recombinant proteins with a final concentration of 100 μg/ml and incubated for 72 h at 37 °C in a 5% CO_2_ incubator. The concentration of extracellular cytokine Interleukin 4 (IL-4) and Interferon gamma (IFN-γ) were determined using ELISA kits (BioLegend, USA) according to the manufacturer’s protocol. The sensitivity limit for the ELISA was deduced from the standard curves after serial dilution of the recombinant mouse IFN-γ and IL-4 standards provided by the kit. The optical density was determined at 450 nm using a microplate reader (Bio-Tek EL × 808, USA). The sensitivity limit of the IFN-γ ELISA was 8 pg / ml and the test interval was 2000 pg / ml-31.3 pg / ml by manufacturer. The sensitivity limit of the IL-4 ELISA was 0.5 pg / ml and the test interval was 125 pg / ml-2 pg / ml.

### Determination of IL-4 secreting CD4^+^, IFN-γ secreting CD4^+^ and CD8^+^ T cells

Single cell suspensions of splenocytes were prepared as described above in Determination of extracellular cytokine by ELISA section. During the last 4 h of incubation, Monensin (BD Biosciences, USA) was added to the cultures at a final concentration of 2 μM according to the manufacturer’s protocol.

T cell populations of control and vaccines groups were simultaneously stained for Alexa flour 647 conjugated rat anti-mouse CD3 (BD Biosciences, USA), FITC conjugated rat *anti*-mouse CD4 (BD Biosciences, USA), or FITC conjugated rat *anti*-mouse CD8a (BD Biosciences, USA) using Cytofix/Cytoperm Plus Fixation/Permeabilization kit (BD Biosciences, USA) according to the manufacturer’s instructions.

Thereafter, the cells were permeabilized and labelled with PE conjugated rat anti-mouse IFN-γ (BD Biosciences, USA) or PE conjugated rat anti-mouse IL-4 antibodies (BD Biosciences, USA) according to the manufacturer’s recommendations. Antibodies were diluted in Perm/Wash solution (BD Biosciences, USA) for intracellular staining. All antibodies were used at a final concentration of 0.5 μg/10^6^ cells.

T cell populations of 10^4^cells, gated with CD3^+^ positive expression, were analysed to quantify: the percentage of rH2, rA4, rE4, rD6, rE6 and rH6 proteins specific CD8^+^ T lymphocytes secreting IFN-γ and CD4^+^ T lymphocytes that secreted IL-4 and IFN-γ using FACSDiva software (BD Biosciences, USA). All data were obtained on a BD FACSAria Flow Cytometer (FACSAria; BD Biosciences, USA).

### Statistical analysis

Data obtained during the experiments were processed using Microsoft Excel Software 2010 and Prism 3.03 program (GraphPad, San Diego, CA). A two-tailed unpaired *t*-test with 95% confidence interval was used to determine the significance between the vaccination groups. Kaplan-Meier survival curves were constructed to illustrate protection from lethal toxoplasmosis. Humoral and cellular immune responses and survival time were expressed as mean ± standard deviation (S.D.).

## Results

### Selection of vaccine candidate antigens using expression levels and bioinformatics analysis

The expression of 49 recombinant proteins were induced with 0.5 mM IPTG when growing cells reached an absorbance of 0.4 ng/μL at OD 600 nm. The cell cultures were harvested 4 h after induction, homogenized with lysis buffer and purified by Ni-NTA beads. Then, the recombinant protein expression levels were assessed by Western blotting and rH2 (TGME49_019310_8), rA4 (TGME49_058660_1), rE4 (TGME49_073130_2), rD6 (TGME49_055180_7), rE6 (TGME49_025340_13), and rH6 (TGME49_013390_5) had detectable bands at 26.7 kDa, 33.9 kDa, 38.8 kDa, 78.1 kDa, 81.2 kDa, and 110.2 kDa, respectively.

**Fig. 1:**
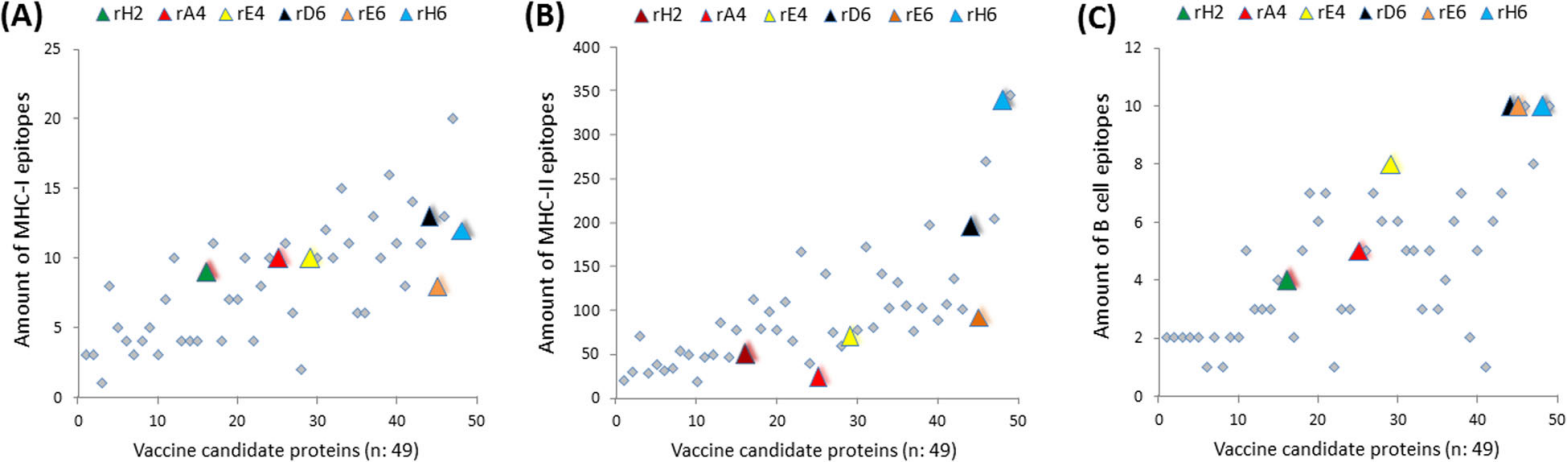
Plot chart showing the amount of **a** MHC-I **b** MHC-II and **c** B cell epitopes in 49 vaccine candidate antigens

Bioinformatic analyses to predict MHC-I, MHC-II, and B cell epitopes of the 49 recombinant proteins using Immune Epitope Database and Analysis Resource (IEDB) and SVMTriP showed that there were 13, 8, 12, 10, 10, and 9 predicted MHC-I epitopes (Fig. 1a) (Table 3), 196, 92, 340, 70, 24, and 50 predicted MHC-II epitopes (Fig. 1b) (Table 3) and 10, 10, 10, 8, 5, and 4 predicted B-cell epitopes (Fig. 1c) (Table 3) in rE6, rD6, rH6, rE4, rA4 and rH2, respectively.

**Table 3:** The amount of MHC-I, MHC-II, and B cell epitopes as well as N-glycosylation and O-glycosylation sites in rD6, rE6, rH6, rE4, rA4 and rH2, and glycosylation sites inside epitopes

Protein	Amino acid content	Amount of MHC-I epitopes in whole protein	Amount of MHC-II epitopes in whole protein	Amount of B cell epitopes in whole protein	Amount of N-glycosylation in whole protein/percent (%)	Amount of O-glycosylation in whole protein/percent (%)	Amount of N-glycosylation in MHC-I epitopes	Amount of O-glycosylation in MHC-I epitopes	Amount of N-glycosylation in MHC-II epitopes	Amount of O-glycosylation in MHC-II epitopes	Amount of N-glycosylation in B cell epitopes	Amount of O-glycosylation in B cell epitopes
**E6**	727	13	196	10	3 (0.41)	81 (7.70)	1	7	3	36	0	18
**D6**	723	8	92	10	2 (0.28)	69 (9.54)	0	4	0	19	0	18
**H6**	1052	12	340	10	2 (0.19)	53 (7.29)	1	5	1	53	0	10
**E4**	364	10	70	8	0 (0.00)	21 (5.77)	0	7	0	5	0	9
**A4**	313	10	24	5	2 (0.64)	6 (1.92)	0	0	0	0	0	2
**H2**	236	9	50	4	0 (0.00)	4 (1.69)	0	0	0	3	0	0

**Fig. 2:**
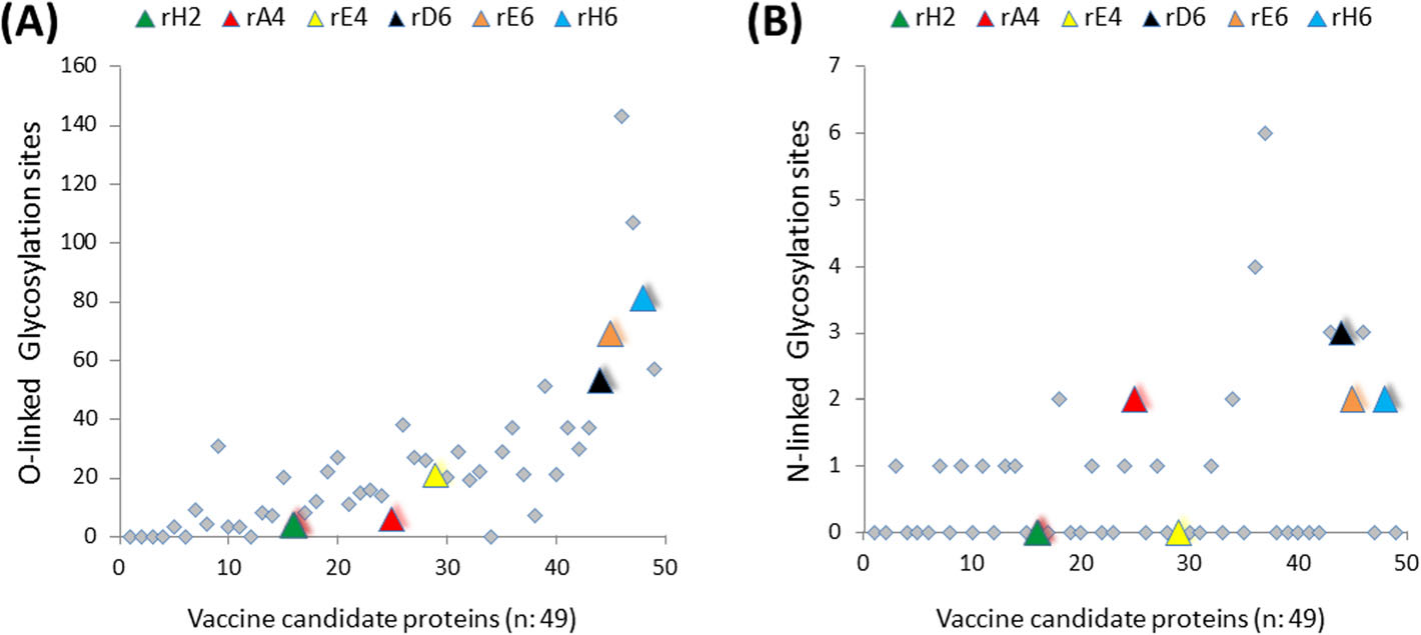
Plot chart showing the **a** O-glycosylation and **b** N-glycosylation sites amount in 49 vaccine candidate antigens

The N-glycosylation and O-glycosylation sites of 49 antigenic proteins were predicted using NetNGlyc 1.0 Server and NetOGlyc 4.0 Server. Moreover, N and O-glycosylation sites on the predicted MHC-I, MHC-II, and B-cell epitopes were also analysed (Table 3). The results showed that rH6, rE6, rD6, rE4, rA4, and rH2 have 81 (7.7%), 69 (9.54%), 53 (7.29%), 21 (5.77%), 6 (1.92%), and 4 (1.69%) O-glycosylation sites, respectively (Fig. 2a) (Table 3). Regarding N-glycosylation, rD6, rA4, rE6 and rH6 proteins have 3 (0.41%), 2 (0.64%), 2 (0.28%), 2 (0.19%) sites respectively, while rH2 and rE4 proteins didn’t have N-glycosylation site (Fig. 2b) (Table 3).

Next, we next focused on the potential glycosylation sites inside predicted MHC-I, MHC-II, and B cell epitopes of rE6, rD6, rH6, rE4, rA4, and rH2. The results showed that in the MHC-I epitopes, rE6 and rH6 have only one N-glycosylation site, rE4, rE6, rH6, and rD6 have 7, 7, 5 and 4 O-glycosylation sites, respectively (Table 3). In MHC-II epitopes, rE6 and rH6 have 3 and one N-glycosylation site, rE6, rD6, rH6, rE4, and rH2 have 36, 19, 53, 5, and 3 O-glycosylation sites, respectively (Table 3). On the other hand, B-cell epitopes of rE6, rD6, rH6, rE4, and rA4 have 18, 18, 10, 9 and 2 O-glycosylation sites but did not contain any N-linked glycosylation site (Table 3).

### Expression and purification of vaccine candidate 6 antigenic proteins

**Fig. 3:**
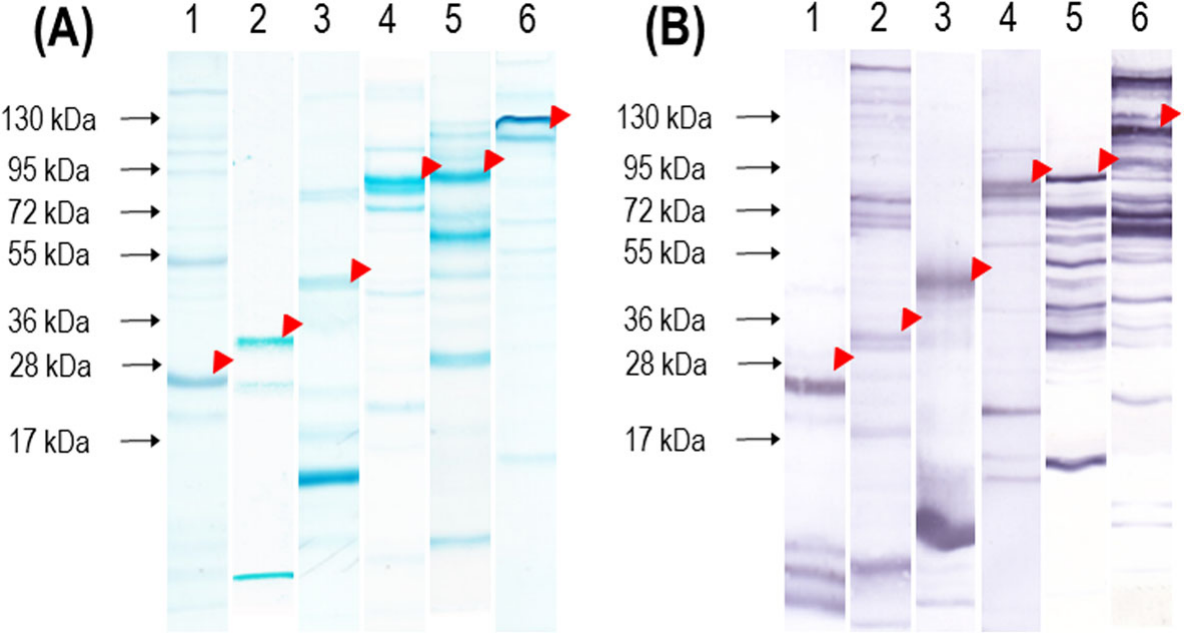
**a** SDS-PAGE and **b** Western blot showing the purified 6 vaccine candidates. Lane 1: rH2 (~ 26.7 kDa); Lane 2: rA4 (~ 33.9 kDa); Lane 3: rE4 (~ 38.8 kDa); Lane 4: rD6 (~ 78.1 kDa); Lane 5: rE6 (~ 81.2 kDa); Lane 6: rH6 (~ 110.2 kDa). Arrowheads show the recombinant proteins

Based on the protein expression levels and MHC-I, MHC-II, and B cell epitope prediction results, rH2, rA4, rE4, rD6, rE6, and rH6 were grown in big batches of LB using a bioreactor, purified in on a HiTrap Ni2+ chelating column and then polished on a gel filtration column to remove excess endotoxin. The purity of rH2, rA4, rE4, rD6, rE6 and rH6 were assessed by SDS-PAGE and western blotting as shown in Fig. 3a and b. The purified rH2, rA4, rE4, rD6, rE6 and rH6 had apparent molecular weight of approximately 26.7 kDa, 33.9 kDa, 38.8 kDa, 78.1 kDa, 81.2 kDa, and 110.2 kDa, respectively. Some of the proteins gave several bands possibly due to multimerization of recombinant protein, degradation or stalling of protein synthesis in *E. coli*.

### Humoral immune response

**Fig. 4:**
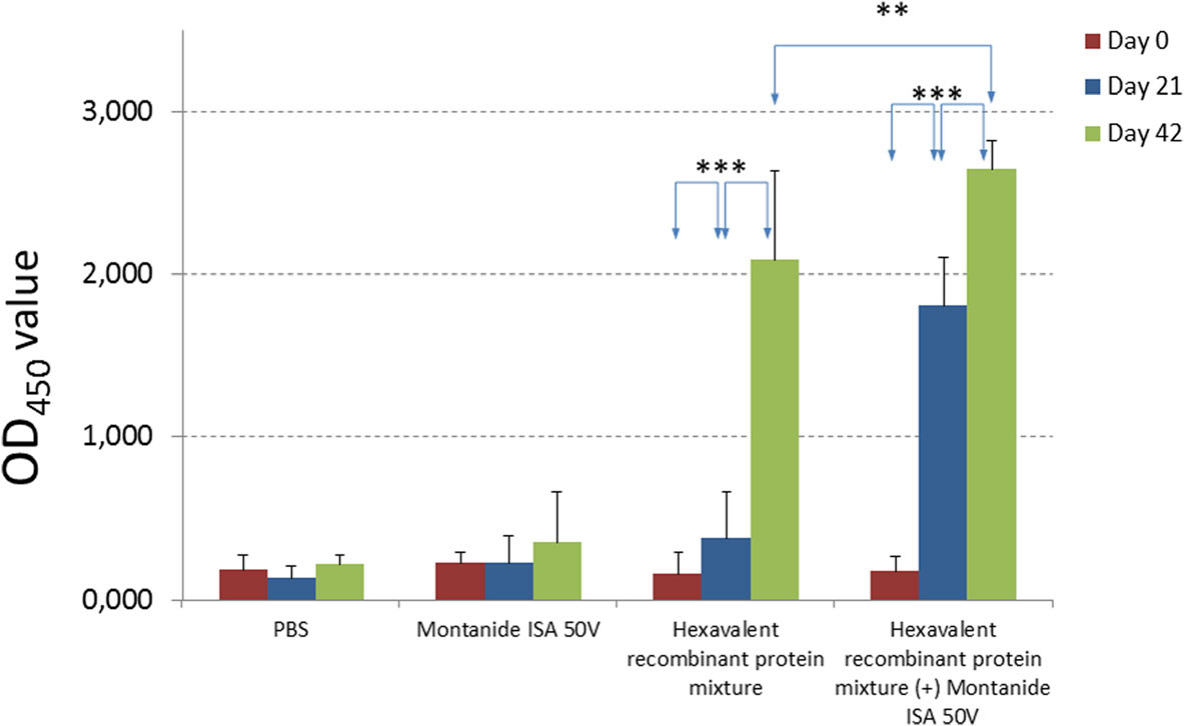
The total IgG response induced by Hexavalent recombinant protein mixture (+) Montanide ISA 50 V and control groups. Each bar represents the mean ± SD value of Total IgG responses of 11 mice from each group. In figure, *** represent *P* ≤ 0.001 and ** represent *P* ≤ 0.01

To determine total IgG, IgG1 and IGg2a antibodies against rH2, rA4, rE4, rD6, rE6, and rH6 proteins in adjuvanted and control group mice serum samples, Rec-ELISA was performed. According to the results, total IgG response detected at day 42 was significantly higher in sera of mice administered with hexavalent recombinant protein mixture (+) Montanide ISA 50 V vaccine and the control group administered with only hexavalent recombinant protein mixture compared to the pre-vaccination sera (*P* < 0.0001, ***). In control groups administered with PBS or Montanide ISA 50 V didn’t induce a significant total IgG response. Significantly high levels of total IgG immune response was detected in the Hexavalent recombinant protein mixture (+) Montanide ISA 50 V when compared with the mice administered with Hexavalent recombinant protein mixture (*P* = 0.002, **) (Fig. 4).

**Fig. 5:**
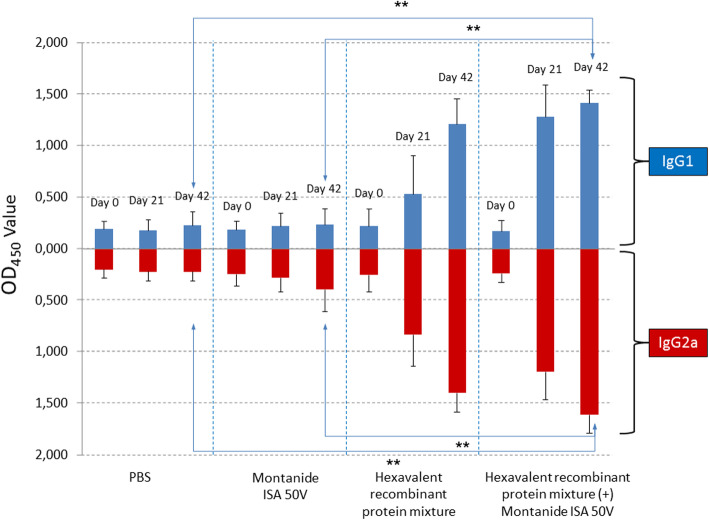
Rec-ELISA analysis of IgG1 and IgG2a subclass antibodies elicited by Hexavalent recombinant protein mixture (+) Montanide ISA 50 V and control groups. The red bars represent IgG2a response and the blue bars, IgG1 response. Each bar represents the mean ± SD value of IgG2a and IgG1 responses of 11 mice from each group. Each bar represents the mean ± SD value of IgG2a and IgG1 responses of 11 mice from each group. In figure, ** represent *P* ≤ 0.01

To assess the polarization of IgG1/IgG2a which is a preliminary marker of whether vaccine induced the Th1 or Th2 immune response, the IgG1 and IgG2a response was analysed by Rec-ELISA. The polarization of IgG1 and IgG2a response is shown in Fig. 5. Hexavalent recombinant protein mixture (+) Montanide ISA 50 V vaccine induced significantly high levels of IgG1 and IgG2a at day 42 compared to the controls groups (*P* < 0.001, **). Overall, in mice administered with Hexavalent recombinant protein mixture (+) Montanide ISA 50 V, IgG2a and IgG1 levels showed a strong and balanced immune response. In control groups, PBS or Montanide ISA 50 V didn’t induce a significant IgG1 or IgG2a response. (Fig. 5).

### Cellular immune response

**Fig. 6:**
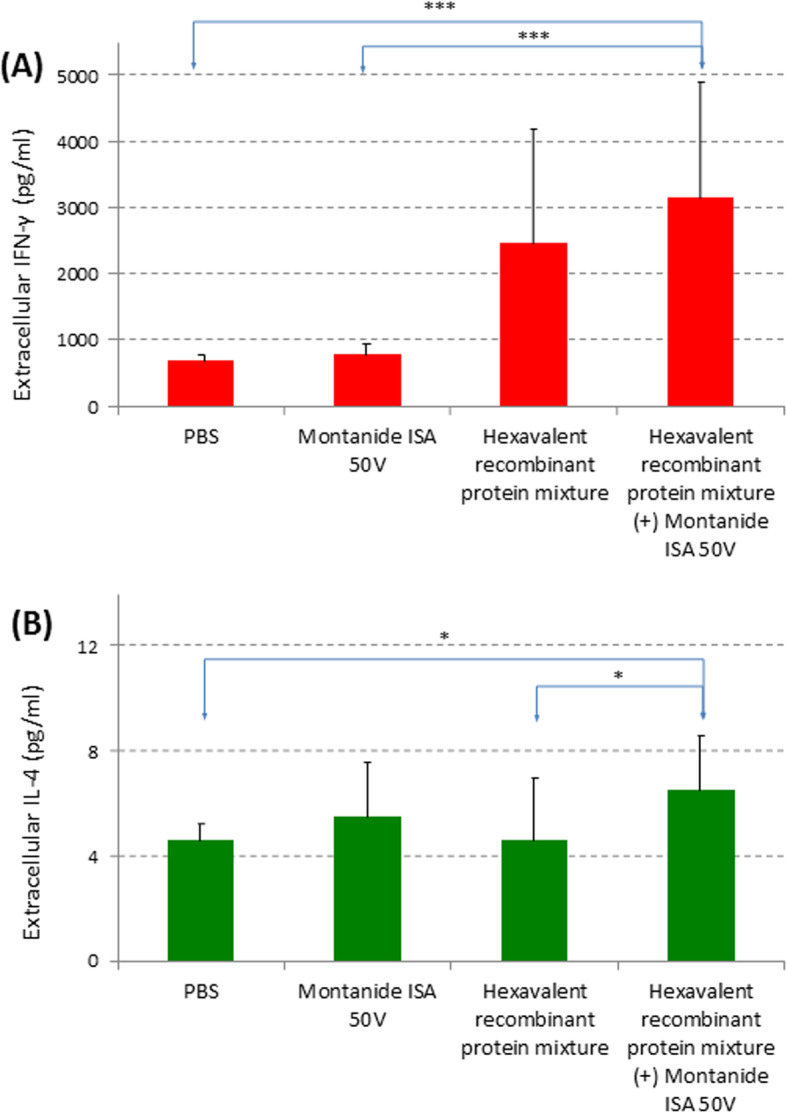
Extracellular **a** IFN-γ and **b** IL-4 levels elicited by hexavalent recombinant protein mixture (+) Montanide ISA 50 V and control groups. The red bars represent IFN-γ response and the green bars, IL-4 response. Each bar represents the mean ± SD value of IFN-γ and IL-4 responses of 3 mice from each group. Each bar represents the mean ± SD value of IFN-γ and IL-4 responses of 3 mice from each group. Single cell suspensions were stimulated with rH2, rA4, rE4, rD6, rE6, and rH6 recombinant proteins with a final concentration of 100 μg/ml. In figure, *** represent *P* ≤ 0.001 and * represent *P* ≤ 0.05

Single-cell suspensions of splenocytes obtained from mice administered with hexavalent recombinant protein mixture (+) Montanide ISA 50 V and controls were stimulated with purified rH2, rA4, rE4, rD6, rE6, and rH6 proteins. Concentration of extracellular cytokine IL-4 and IFN-γ were determined using ELISA. According to the results, IFN-γ level was significantly higher in mice vaccinated with the hexavalent recombinant protein mixture (+) Montanide ISA 50 V compared to mice vaccinated with only Montanide ISA 50 V (*P* = 0.001,***) or PBS (*P* = 0.0007,***) (Fig. 6a). On the other side, IL-4 level was also significantly high in mice vaccinated with the hexavalent recombinant protein mixture (+) Montanide ISA 50 V compared to control groups administered with PBS (*P* = 0.0133, *) or Hexavalent recombinant protein mixture (*P* = 0.0183, *) (Fig. 6b).

**Fig. 7:**
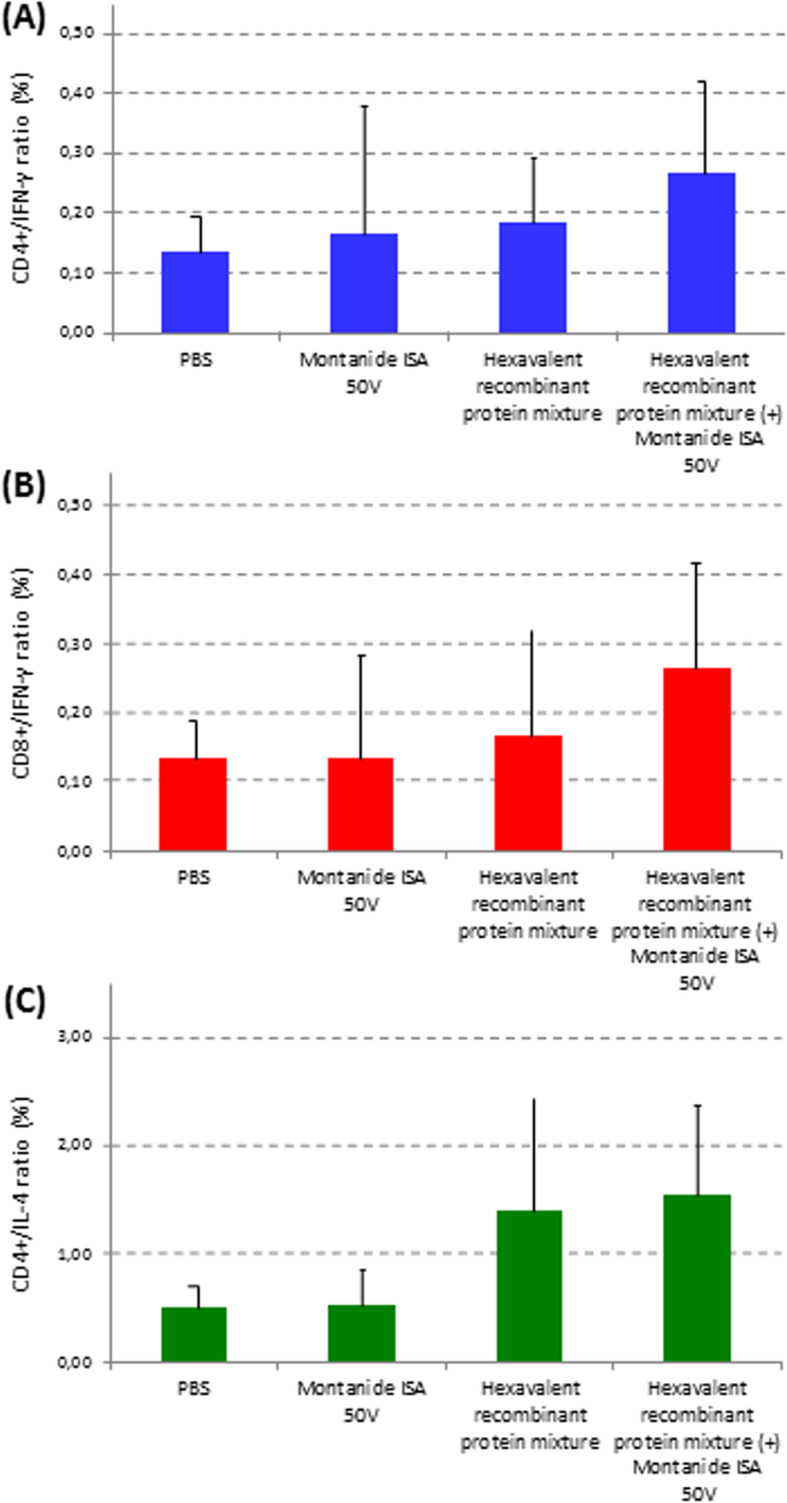
**a** The ratio of CD4+ T lymphocytes secreting IFN-γ **b** the ratio of CD8+ T lymphocytes secreting IFN- γ **(C)** the ratio of CD4+ T lymphocytes secreting IL-4 obtained by vaccinating mice with hexavalent recombinant protein mixture (+) Montanide ISA 50 V and control groups. The percentage values represent the amount of CD8+ and CD4+ T lymphocytes secreting IFN-γ and CD4+ T lymphocytes secreting IL-4 in 10^4^ T cell population gated with CD3+ positive expression

Flow cytometry analysis was used to determine the ratio of CD8^+^ T lymphocytes secreting IFN-γ and CD4^+^ secreting IFN-γ and IL-4 in vaccinated and control groups. Protection against intracellular parasite like *T. gondii* is primarily achieved by CD8^+^ T lymphocytes secreting IFN-γ. For this reason, CD8^+^ T cell response is important for an ideal vaccine against toxoplasmosis [11, 26, 27]. T cell population has been gated with CD3^+^ staining. In mice vaccinated with Hexavalent recombinant protein mixture (+) Montanide ISA 50 V vaccine, the ratio of CD4^+^ T lymphocytes secreting IFN-γ increased 2, 1.6 and 1.45 times compared to control groups vaccinated with PBS, Montanide ISA 50 V, and only Hexavalent recombinant protein mixture (Fig. 7a). Besides, the ratio of CD8^+^ T lymphocytes secreting IFN-γ increased 2.2 and 1.6 times in mice vaccinated with Hexavalent recombinant protein mixture (+) Montanide ISA 50 V compared to control groups vaccinated with PBS, Montanide ISA 50 V, and only Hexavalent recombinant protein mixture (Fig. 7b). On the other hand, the ratio of CD4^+^ T lymphocytes secreting IL-4 in mice vaccinated with Hexavalent recombinant protein mixture (+) Montanide ISA 50 V increased 3.07, 2.88, and 1.1 times compared to control groups vaccinated with PBS, Montanide ISA 50 V, and only Hexavalent recombinant protein mixture (Fig. 7c). CD3^+^ T cells from all experimental groups proliferated to comparable ratios in response to ConA (Data not shown).

### Protective effect of mice immunized against challenge with lethal *T. gondii*

**Fig. 8:**
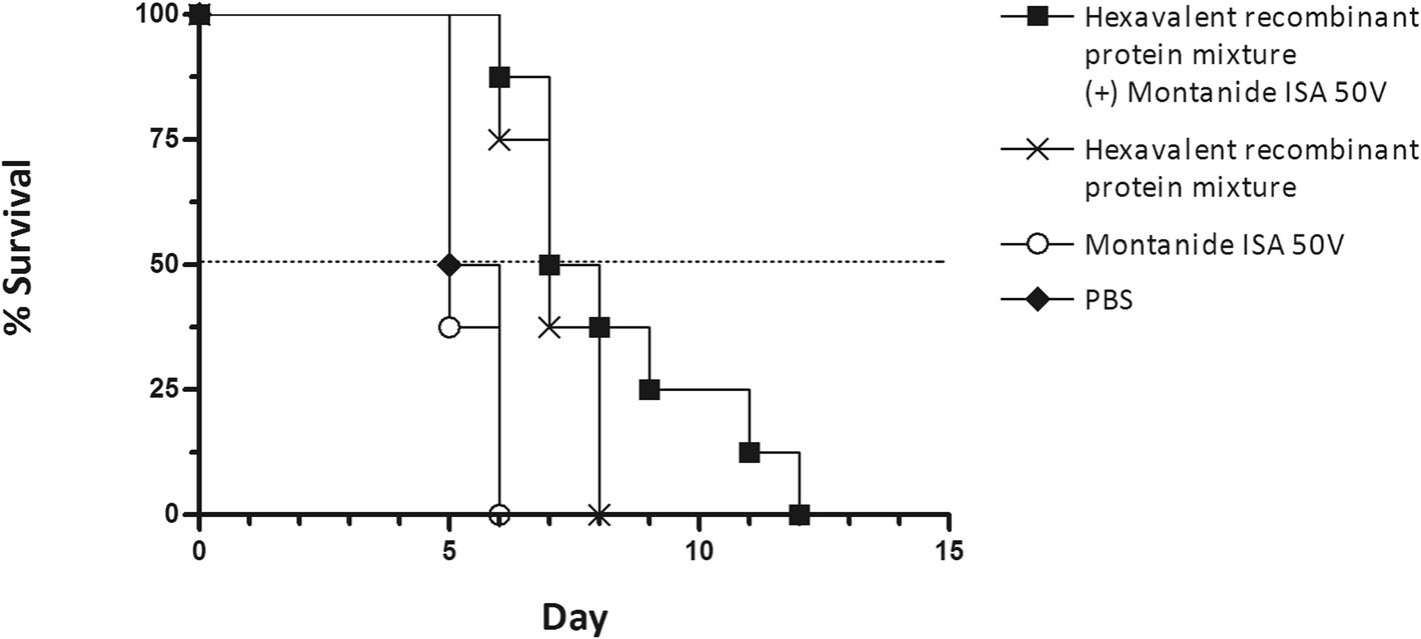
Survival profiles of vaccinated mice against lethal toxoplasmosis. Eight mice from all four groups were challenged with 1 × 10^5^*T. gondii* Ankara strain tachyzoites nine weeks after the prime vaccination. *T. gondii* tachyzoites were administered to mice vaccinated with hexavalent recombinant protein mixture (+) Montanide ISA 50 V (■), and the control groups hexavalent recombinant protein mixture (×), Montanide ISA 50 V (○), and PBS (♦)

Protection against lethal toxoplasmosis in mice vaccinated with Hexavalent recombinant protein mixture (+) Montanide ISA 50 V was determined using *T. gondii* Ankara strain tachyzoites. Eight from each mice group were administered intraperioneally with 1 × 10^5^*T. gondii* Ankara strain tachyzoites 9 weeks after the prime vaccination. After challenging, mice groups administered with PBS and only Montanide ISA 50 V survived 5.5 ± 0.53 and 5.38 ± 0.52 days, respectively. Mice vaccinated with Hexavalent recombinant protein mixture and Hexavalent recombinant protein mixture (+) Montanide ISA 50 V survived approximately 7. ±0.83 days and 8.38 ± 2.13, respectively. Survival was significantly prolonged in groups vaccinated with Hexavalent recombinant protein mixture and Hexavalent recombinant protein mixture (+) Montanide ISA 50 V compared to controls (*P* < 0.01). The results are summarized in Fig. 8.

## Discussion

Recently, our study group has discovered some *T. gondii* proteins that can be used as vaccine candidate against toxoplasmosis using in silico and immunoscreening approaches based on protein microarrays [6, 8]. We selected the antigens from this screening approach because antigens selected without a screening approach ended with disappointing clinical results such as the malaria vaccine RTS,S which is a hybrid protein particle designed in 1980s. The Major Surface Glycoprotein (MSG) containing hybrid protein was formulated in a multi-component adjuvant (AS01) and showed 39% protection in East African children in 2011 [28]. For these reasons, development of a multivalant recombinant protein vaccine using some of these discovered proteins was the aim of this study.

For this purpose, we used the data from protein microarray screening and prioritized 49 proteins based on their immunogenicity. To further analyse these proteins for their availability for vaccine development against toxoplasmosis, we conducted small scale protein expression experiments in conjunction with bioinformatics analyses. According to protein expression levels 6 proteins were suitable to be used in the vaccine development. Among them, rH2 (TGME49_019310_8) is a DnaK family protein, rA4 (TGME49_058660_1) is ROP6, rE4 (TGME49_073130_2) is a SAG-related sequence SRS30A, rD6 (TGME49_055180_7) is an ubiquitin carboxyl-terminal hydrolase, rE6 (TGME49_025340_13) is a hypothetical protein, and rH6 (TGME49_013390_5) is a plectin.

The protection against *T. gondii* infection is dependent on CD8^**+**^ T-cytotoxic lymphocytes which play a significant role in cell mediated protection as well as B cells which is important in humoral immune response [11–13, 15–19]. For this reason, MHC-I, MHC-II, and B-cell epitopes of the vaccine candidate proteins were predicted by bioinformatics. SVMTriP online service was used to analyse the B-cell epitopes of rE6, rD6, rH6, rE4, rA4, and rH2 proteins. As shown in Fig. 1c, the presence of B-cell epitopes on the 6 vaccine candidate proteins suggests that they have a strong potential to act as a B-cell antigen. We also used the online service IEDB to analyse MHC-I and MHC-II epitopes of rE6, rD6, rH6, rE4, rA4, and rH2 proteins and found MHC-I and MHC-II epitopes on the 6 vaccine candidate proteins (Fig. 1a and b).

Protein glycosylation has great importance in terms of protein stability, three-dimensional structure, surface expression, activity, and antigenicity [29]. Previously, it was shown that *T. gondii* contains 11 N-linked glycosylated proteins and 15 O-linked glycosylated proteins by lectin-probed western blot analysis. Moreover, it is reported that N and O-linked glycosylated proteins are found throughout the secretory pathway of the *T. gondii* and N-linked glycosylation of proteins is essential for the survival of parasite [20]. For this reason, N-and O-linked glycosylation sites of 49 antigenic proteins, which may be candidates for vaccination, was predicted by bioinformatics. The results suggest that the 6 vaccine candidate proteins contain N-linked glycosylation sites except for the rH2 and rE4 proteins (Fig. 2b). O-linked glycosylation sites are found on rE6, rD6, rH6, rE4, rA4, and rH2 proteins (Fig. 2a).

At this stage, we further examined the N- and O-linked glycosylation sites in B cell, MHC-I, and MHC-II epitopes of the 6 vaccine candidate antigens by bioinformatics. The results demonstrate that B-cell epitopes of 6 vaccine candidate proteins were not containing N-linked glycosylation sites. B-cell epitopes of rE6, rD6, rH6, rE4, rA4 were O-glycosylated (Table 3). The N-linked glycosylation site in the MHC-I epitopes were only found in the rE6 and rH6 proteins and the O-linked glycosylation is detected in the rE4, rE6, rH6, and rD6 proteins (Table 3). The N-linked glycosylation site in the MHC-II epitopes were similarly found in the rE6 and rH6 proteins and the O-linked glycosylation is detected in the rE6, rD6, rH6, rE4, and rH2 proteins (Table 3). These results show that the 6 vaccine candidate proteins and their epitopes are glycosylated at various ratios and their antigenicity is high.

Thereafter, we developed a hexavalent recombinant protein protein vaccine adjuvanted with Montanide ISA 50 V which has shown to induce strong cellular and humoral immune response. After vaccination of *Swiss Webster outbred* mice, strong total IgG, IgG1 and IgG2a responses were detected in mice administered with hexavalent recombinant protein mixture (+) Montanide ISA 50 V compared to control groups vaccinated with only Montanide ISA 50 V or PBS (*P* < 0.0001) indicating strong and balanced Th1 and Th2 responses (Figs. 4 and 5). The production of extracellular IFN-γ was significantly higher in mice vaccinated with the hexavalent recombinant protein mixture (+) Montanide ISA 50 V compared to mice vaccinated with only Montanide ISA 50 V or PBS (*P* < 0.001) (Fig. 6a). Flow cytometry results were also in compatible with extracellular ELISA in which hexavalent recombinant protein mixture (+) Montanide ISA 50 V showed increment in ratio of CD8^+^ and CD4^+^ T lymphocytes secreting IFN-γ (Fig. 7a and b).

CD4+ T lymphocytes secreting IL-4 were also increased according to flow cytometry results as well as there was an increase in extracellular IL-4 secretion according to ELISA (Figs. 6b and 7c). Flow cytometry results, specifically CD4^+^ T lymphocytes secreting IL-4 cell ratio is bigger than CD4^+^ T lymphocytes secreting IFN-γ which contradict with ELISA results in which IFN-γ was higher than IL-4 secretion. This discrepancy can interpreted as IFN-γ levels detected by ELISA can be related to CD8^+^ T lymphocytes secreting and macrophages other than CD4^+^ T lymphocytes. On the other side, the main protective cells against toxoplasmosis are conferred by CD8^+^T lymphocytes secreting IFN-γ which has increased with recombinant protein mixture (+) Montanide ISA 50 V compared to controls. The protective efficacy of hexavalent recombinant protein mixture (+) Montanide ISA 50 V against lethal toxoplasmosis was evaluated by infecting mice intraperitoneally with *T. gondii* Ankara strain tachyzoites. *T. gondii* Ankara strain is Africa 1 genotype and causes death in mice in approximately 4–5 days [22]. Challenging study showed that survival was prolonged from 4 to 5 days which was observed in control group mice administered with only Montanide ISA 50 Vand PBS to more than 11 days in two mice vaccinated with hexavalent recombinant protein mixture (+) Montanide ISA 50 V (Fig. 8).

In this study, Montanide ISA 50 V was selected as an adjuvant due to its efficiency in inducing both humoral and cellular immune responses. Montanide ISA 50 V was used as adjuvant in previous studies against Bovine herpesvirus 5, *Boophilus microplus*, Foot and Mouth Disease Virus (FMDV), and *Leishmania major* [30–33]. In the vaccine trial against FMDV, *Boophilus microplus,* and *Leishmania major*, Montanide ISA 50 V induced significant levels of protective cytokine production and/or antibody response. During the vaccine trial with recombinant glycoprotein D of Bovine herpesvirus 5, a mixed Th1/Th2 response was elicited [30]. In this study, hexavalent recombinant protein mixture (+) Montanide ISA 50 V showed strong and balanced Th1 and Th2 responses. Overall, the Th1 part of the immune response elicited by hexavalent recombinant protein mixture (+) Montanide ISA 50 V induced significant levels of CD4^+^ and CD8^+^ T lymphocytes secreting IFN-γ and conferred significant protection in Swiss Outbred mice challenged with lethal dose of *T. gondii* Ankara strain tachyzoites.

In literature, multivalent recombinant protein vaccines have been developed against toxoplasmosis. In these studies, surface related antigens rSAG1, rSAG2, rSAG3, rSRS1, rP54, rSRS4, and rSRS9; dense granule proteins rGRA1, rGRA2, rGRA4, rGRA5, rGRA6, and rGRA7; rhoptry proteins rROP2, rROP4, and rROP5 as well as rTgPI-1, rMAG1 and rBAG1 have been used [24, 34–44].

In all of the recombinant protein or DNA vaccine studies against toxoplasmosis, murine models are being used to determine the immunity and protection conferred by vaccinations. Michima et al., 2001 vaccinated mice with a mixture of rSAG1, rSAG2, rSAG3, rSRS1, and rP54 proteins adjuvanted with Freund and achieved 17% survival up to 120 days after i.p. challenging with *T. gondii* Beverley strain bradyzoites [34]. Golkar et al., 2007 vaccinated mice with a mixture of rGRA2 and rGRA6 proteins adjuvanted with monophosphoryl lipid A and achieved 48.2% decrease in brain cyst formation after i.p. challenging with *T. gondii* Pru strain cysts [35]. In one study, rROP2, rGRA5, rGRA7 proteins and cholera toxin were administered through intranasal route to mice and oral challenge with VEG cysts resulted in 58.3% decrease in brain cysts compared to controls [36]. In a study that used antigenic epitopes of SAG1, GRA1, and MAG1 proteins adjuvanted with Freund decreased the brain cysts formation by 89% [37]. Dziadek et al., 2011, evaluated rROP2, rROP4, rGRA4, and rSAG1 in three vaccine formulations adjuvanted with incomplete Freund administered subcutaneously to mice and challenge with *T. gondii* DX cysts results with 71 to 90% decrease in parasite burden [38]. In another study, a synthetic peptide, generated using B-cell and two T-cell epitopes derived from SAG1, GRA4, and GRA1 antigens was adjuvanted with Freund and challenge with GJS tachyzoites increased survival time [39]. In other study, mice were vaccinated with the mixture of rROP5 and rSAG1 adjuvanted with Freund and challenged with the *T. gondii* RH strain. The results showed that *T. gondii*-specific IgG antibodies levels and lymphocyte proliferative responses are increased in vaccine group and conferred more efficient protection compared to the control groups [40]. Sun et al., 2014 vaccinated mice with a mixture of rBAG1, rSRS4, and rSRS9 proteins adjuvanted with Freund or recombinant mindin. The results showed that vaccine using mindin as an adjuvant efficiently stimulated humoral and cellular responses, including antigen-specific IgG1 and IgG2a, as well as lymphocyte proliferation. Also the improved protection against *T. gondii* infection was observed in the mindin adjuvanted vaccine group compared with the other controls [41]. In another study, T- and B-cells epitopes of AMA1, RON2, and RON4 proteins was used to develop multivalent peptide vaccine formulations. The IgA levels were increased in the mice immunized with single rRON2, while the IgG levels were higher in the mice immunized with rAMA1 (+) rRON. Significant level of IFN-γ was detected in mice immunized with rAMA1 (+) rRON2. Infection with *T. gondii* is naturally occurs through the oral route by water or food contaminated with tissue cysts (containing bradyzoites) or oocysts (containing sporozoites). Interestingly, in this study, the mice were challenged orally with 4 × 10^4^ tachyzoites of *T. gondii* RH strain. It was reported that the control mice died within 13 days. The mice immunized with A1 + R2 achieved 70% survival rates. The mice immunized with AMA1, A1 + R2 + R4, and RON2 achieved 60, 50, and 40% survival rates, respectively [42]. Another vaccine trial using T- and B-cells epitopes of SAG1, GRA2, GRA7, and ROP16 proteins resulted with higher levels of IgG and IgG2a subclass titters, significant production of IFN-γ, percentage of T lymphocyte subsets and longer survival times against intraperitoneal challenge with 10^3^*T. gondii* RH strain tachyzoites [43].

rGRA1 and rBAG1 were used in a multivalent recombinant protein vaccine adjuvanted with Alum. Significant increase in IgG, IgG2a subclass titters as well as significant increment in ratio of IFN-γ secreting CD4^+^ and CD8^+^ T lymphocyte subsets were achieved. Mice were challenged orally with 10–12 *T. gondii* PRU strain tissue cysts and the amount of tissue cysts in vaccinated group decreased 10.5% compared to control groups. In sum, multistage and multivalent rBAG1 and rGRA1 vaccine increased immune response but induced partial protection against toxoplasmosis [24].

Picchio et al., 2018 vaccinated mice with rTgPI-1, rROP2, and rGRA4 proteins ajuvanted with Alum intradermally or with CpG-ODN 1826 intranasally and mice were orally challenged with the *T. gondii* ME49tissue cyst. P + R + G vaccine formulations induced significant decreases in the number of cysts per brain compared to the control group. According to the levels of IgG1 and IgG2a subclasses P + R + G vaccine groups showed a mixed Th1/Th2 immunity [44].

## Conclusions

Overall, in the present study a hexavalent recombinant protein vaccine adjuvanted with Montanide ISA 50 was first time developed and administered to mice to protect against lethal toxoplamosis. Moreover, the immunogenic and protective efficiency of rROP6, DnaK family protein, SRS30A, ubiquitin carboxyl-terminal hydrolase, and plectin was first time tested in an animal model. In addition, Montanide ISA 50 V was first time used as an adjuvant in mice model against toxoplasmosis. Apart from these, multiplexing recombinant proteins induced strong and balanced Th1 and Th2 immune responses and improved protection against toxoplasmosis and thus showing the the importance of using multivalant recombinant protein vaccines in future vaccine development studies against ruminants, cats or humans.

## Data Availability

The dataset analyzed during the current study is available from the corresponding author on reasonable request.

## Abbreviations

*T. gondii*: *Toxoplasma gondii*
*E. coli*: *Escherichia coli*
OD: Optical density
IPTG: Isopropyl-D- thiogalactopyranoside
rpm: Rounds per minute
Ni-NTA: Nickel-charged affinity resin
MHC: Major histocompatibility complex
IEDB: Immune Epitope Database and Analysis Resource
SVMTriP: Support Vector Machine Tri-peptide similarity and Propensity scores
FPLC: Fast Protein Liquid Chromatography
SDS-PAGE: Sodium dodecyl sulfate-polyacrylamide gel electrophoresis
PVDF: Polyvinylidene difluoride
BCIP: 5-bromo-4-chloro-3-indolyl phosphate
LAL: Limulus Ameobocyte Lysate
EU: Endotoxin Units
i.p.: Intraperitoneally
IgG: Immune globulin G
IgG1: Immune globulin G1
IgG2a: Immune globulin G2a
Rec-ELISA: Recombinant Enzyme-Linked ImmunoSorbent Assay
PBS: Phosphate buffered saline
TMB: 3, 3′, 5, 5′ tetramethylbenzidine
AV: Absorbance value
S.D.: Standard deviation
FCS: Fetal calf serum
IL-4: Interleukin 4
IFN-γ: Interferon gamma
FMDV: Foot and Mouth Disease Virus
MWCO: Molecular weight cut-off
